# Non-Antibiotic Prophylaxis for Urinary Tract Infections

**DOI:** 10.3390/pathogens5020036

**Published:** 2016-04-16

**Authors:** Mariëlle Beerepoot, Suzanne Geerlings

**Affiliations:** Department of Internal Medicine, Division of Infectious Diseases, Academic Medical Center, Meibergdreef 9, 1105 AZ Amsterdam, The Netherlands; S.E.Geerlings@amc.uva.nl

**Keywords:** urinary tract infections, prevention and control, review, non-antibiotic strategies

## Abstract

Increasing antimicrobial resistance has stimulated interest in non-antibiotic prophylaxis of recurrent urinary tract infections (UTIs). Well-known steps in the pathogenesis of UTIs are urogenital colonization and adherence of uropathogens to uroepithelial cell receptors. To prevent colonization in postmenopausal women, vaginal, but not oral, estrogens have been shown to restore the vagina lactobacilli flora, reduce vaginal colonization with *Enterobacteriaceae,* and reduce the number of UTIs compared to placebo. Different lactobacilli strains show different results in the prevention of recurrent UTIs. Intravaginal suppositories with *Lactobacillus crispatus* in premenopausal women and oral capsules with *Lactobacillus rhamnosus GR-1* and *Lactobacillus reuteri RC-14* in postmenopausal women are promising. Ascorbic acid (vitamin C) cannot be recommended for the prevention of UTIs. Cranberries are thought to contain proanthocyanidins that can inhibit adherence of P-fimbriated *E. coli* to the uroepithelial cell receptors. Cranberry products decreased UTI recurrences about 30%–40% in premenopausal women with recurrent UTIs, but are less effective than low-dose antimicrobial prophylaxis. However, the optimal dose of cranberry product has still to be determined. Initially OM-89, a vaccine with 18 heat-killed *E. coli* extracts, seemed promising, but this was not confirmed in a recently randomized trial.

## 1. Introduction

Approximately 20%–30% of women with a urinary tract infection (UTI) will have a recurrence. Recurrent UTIs are defined as at least three episodes of a UTI in twelve months, or at least two episodes in six months [1]. Recurrent UTIs can be subdivided into relapses and reinfections. A relapse is defined as a UTI caused by the same microorganism after adequate treatment. Reinfection refers to recurrence of a UTI caused by a different microorganism or a recurrent UTI caused by a previously isolated microorganism after treatment and a subsequent negative urine culture [2]. Reinfections can be managed with daily or every other day low-dose antibiotic prophylaxis, a short course of antibiotics initiated immediately at the onset of symptoms, or postcoital antibiotic prophylaxis [3]. Antibiotics are effective in the treatment of UTIs and for low-dose antibiotic prophylaxis but lead to an increase in antibiotic resistance in microorganisms [4]. In a study with 86 healthy students, 82 of them had resistant microorganisms in their feces after two weeks of trimethoprim (with or without sulfamethoxazole) prophylaxis [5]. In addition, antibiotics can cause side effects. Women with recurrent UTIs are increasingly asking their healthcare professionals about the value of taking non-antibiotic products. Clearly, there is a need to identify strategies that target alternative pathways involved in the pathogenesis of UTIs.

## 2. Pathogenesis of UTIs

### 2.1. Urogenital Colonization

The majority of UTIs are caused by bacteria from the intestine that ascend through the urethra and the bladder and, sometimes, to the kidneys. The first step in the pathogenesis is the colonization of the vaginal introitus and urethral meatus with uropathogens, after which the bladder is colonized through the urethra. The vaginal flora plays an important role in the prevention of UTIs. The lactobacilli-dominated vaginal flora in premenopausal women impedes the colonization of uropathogens due to competitive exclusion and maintaining a low vaginal pH [6]. The main defense mechanisms of the host against bladder colonization are dilution and micturition. Anatomical or functional abnormalities that prevent complete emptying of the bladder increase the likelihood of colonization with uropathogens [7].

### 2.2. Adhesion of Uropathogens

After colonization, the next step in the pathogenesis of a UTI is the adhesion of uropathogens to the epithelial bladder cells. Following adherence, uropathogens are protected from removal by micturition. The adhesion of *E. coli* to the uroepithelial cell receptors of the host is accomplished by hair-like organelles called fimbriae. The most important are Type 1 fimbriae and P-fimbriae. Type 1 fimbriae mainly play a role in the pathogenesis of cystitis and P-fimbriae in pyelonephritis [7]. In women with recurrent UTIs an increased adherence of *E. coli* to urogenital epithelial cells was seen compared to healthy controls [8]. Several studies have suggested that the binding of uropathogenic *E. coli* to epithelial cells is dependent in part on the histo-blood group secretor status [9]. Moreover, the positive correlation between a UTI infection history in first-degree female relatives and UTI risk suggests a genetic component for increased susceptibility [10].

### 2.3. Invasion

While UTIs are typically considered extracellular infections, it has been demonstrated that uropathogenic *E. coli* can invade and replicate within the bladder cells to form intracellular bacterial communities (IBCs) [11]. In women with acute uncomplicated symptomatic UTIs, most commonly caused by uropathogenic *E. coli*, IBCs could be detected in exfoliated urothelial cells in about one fifth of urine specimens [12]. In a recent study in children with an *E. coli* UTI, IBCs were found in exfoliated cells in about one third of the children. The presence of intracellular bacteria was associated with recurrent UTI [13]. These findings are of uncertain clinical significance, but raise the possibility that the presence of IBCs in urine might identify women who would benefit either from longer treatment with antibiotics or treatment with antibiotics that kill intracellular bacteria.

Although an association between IBCs and recurrent UTIs was found, in women with recurrent UTIs asymptomatic bacteriuria was not predictive for the development of a UTI. However, the susceptibility and pulsed-field gel electrophoresis pattern of *E. coli* strains isolated from urine in the month before a symptomatic *E. coli* UTI were similar in about three quarter of patients. These findings suggest an intracellular bacterial reservoir could possibly serve as a nidus for recurrence in same-strain UTIs in women with recurrent UTIs [14].

## 3. Prevention of UTIs

### 3.1. Prevention of Colonization

#### 3.1.1. Estrogens

After menopause only 25% to 30% of women have lactobacilli in the vagina. With estrogen replacement therapy this percentage may increase to 60% to 100% [15]. In a placebo-controlled study with intravaginal estrogens in postmenopausal women with recurrent UTIs, no intravaginal lactobacilli were present at baseline. After one month of treatment, in 22 out of 36 women in the estrogen group intravaginal lactobacilli were present, compared with zero of the 24 women in the placebo group (difference 61%, 95% confidence interval (CI) 45% to 77%). In the estrogen group the vaginal pH decreased from 5.5 to 3.8 (*p* < 0.001), while there was no change in the placebo group. The percentage of women with vaginal colonization with *Enterobacteriaceae* in the estrogen group decreased from 67% to 31%, but was virtually unchanged in the placebo group. The incidence of UTIs was lower in the estrogen group compared to the placebo group: 0.5 *versus* 5.9 episodes per patient year (*p* < 0.001) [16]. In another trial in which women received either an estradiol-releasing ring or no treatment, the vaginal estrogens reduced the proportion of women with a UTI by about one third [17]. Although vaginal estrogens reduced the number of UTIs, oral estrogens did not. In addition, oral estrogens are associated with coronary heart disease, venous thromboembolism, stroke, and breast cancer. Therefore, oral estrogens are not recommended in postmenopausal women to prevent recurrent UTIs [18].

#### 3.1.2. Lactobacilli

The precise interaction of lactobacilli with the commensal flora and the host, and the mechanism of action by which they exert their beneficial effects are still largely unknown. However, specific lactobacilli strains seem to have the ability to interfere with the adherence, growth, and colonization of uropathogenic bacteria [19].

In the study by Baerheim *et al.* [20], 48 women were randomized to vaginal suppositories containing *L. casei v rhamnosus*, twice weekly for 26 weeks, or placebo. The study from Kontiokari *et al.* [21] was an open randomized trial in 150 women who had UTIs caused by *E. coli*. After being treated with antimicrobial agents for the UTI, they were randomly allocated to one of three groups. The first group received 50 mL of cranberry lingonberry juice per day, the second group took 100 mL of a Lactobacillus GG (4 × 10 ^10^ cfu) drink five days per week, and a third group received no further treatment. In both studies lactobacilli prophylaxis did not show an advantage in terms of UTI prevention compared to placebo or no treatment. Baerheim reported a monthly incidence of symptomatic UTI of 0.21 in the *L. casei* group and 0.15 in the placebo group (incidence rate ratio 1.41; 95% CI 0.88–1.98). Kontiokari also did not show a difference between *Lactobacillus* GG drink and no treatment. At 12 months 21 (42.9%) and 19 (38.0%) women in the lactobacillus and control groups, respectively, had a UTI. The mean number of UTIs experienced during the 12-months follow-up was 0.80 UTIs/patient in the lactobacilli-treated women compared to 0.76 UTI/patient in the control group.

In another double-blind placebo-controlled trial, 100 premenopausal women with at least one prior UTI in the last year were randomized to receive *Lactobacillus crispatus* containing intravaginal suppositories or placebo following antimicrobial treatment for an acute UTI. Recurrent UTI occurred in seven of 48 women (15%) receiving Lactin-V compared to 13 out of 48 women (27%) receiving placebo (Relative Risk (RR) 0.5; 95% CI 0.2–1.2). High-level vaginal colonization with *L. crispatus* (≥10^6^ throughout follow-up) was associated with a significant reduction in recurrent UTI only for Lactin-V (RR for Lactin-V 0.07; RR for placebo 1.1; *p* < 0.01) [22].

A trial of antibiotic prophylaxis and lactobacillus prophylaxis was undertaken in 252 postmenopausal women with recurrent UTIs. They were randomized to receive 12 months of prophylaxis with trimethoprim-sulfamethoxazole (TMP-SMX), 480 mg once daily or oral capsules containing 10^9^ colony-forming units of *Lactobacillus rhamnosus* GR-1 and *Lactobacillus reuteri* RC-14 twice daily. The mean number of symptomatic UTIs in the year preceding randomization was 7.0 in the TMP-SMX group and 6.8 in the lactobacilli group. In the intention-to-treat analysis, after 12 months of prophylaxis, these numbers were 2.9 and 3.3, respectively. The between-treatment difference of 0.4 UTIs per year (95% CI, −0.4 to 1.5) was outside the non-inferiority margin of 10%. After one month of TMP-SMX prophylaxis, resistance to TMP-SMX, trimethoprim, and amoxicillin had increased from 20%–40% to 80%–95% in *E. coli* from the feces and urine of asymptomatic women, and among *E. coli* causing a UTI. During the three months after TMP-SMX discontinuation, resistance levels gradually decreased. Resistance did not increase during lactobacilli prophylaxis [23].

#### 3.1.3. Vitamin C (Ascorbic Acid)

Many women use vitamin C to prevent UTIs, but only two trials, with contradictory results, have been reported. In the first study the effect of ascorbic acid on urine pH was studied in spinal cord injury patients. The study was designed to compare the baseline urine pH after the administration of placebo or 500 mg ascorbic acid four times daily. Of the 38 patients who began the study, only 13 completed it. A significant decrease in urine pH value was not observed. There was no clinical benefit from the use of ascorbic acid; two patients in the ascorbic acid and one patient in the placebo group developed a UTI between the sixth and eighth day after initiating the study [24].

The other, non-randomized, trial in 110 pregnant women reported that daily intake of a vitamin regimen with 100 mg ascorbic acid for three months reduced the incidence of symptomatic UTIs from 29.1% to 12.7% compared to a vitamin regimen without ascorbic acid [25]. However, it is difficult to evaluate these results, as the daily vitamin C dose was very low. In addition, it was unclear if a urine culture was done when the women had symptoms of a UTI.

### 3.2. Prevention of Adherence

#### 3.2.1. Cranberries

Cranberries have been used in the prevention of UTIs for many years. The mechanism of action has not been completely elucidated. Based on *in vitro* studies, cranberries are thought to contain proanthocyanidins (PACs) that can inhibit adherence of P-fimbriated *E. coli* to the uroepithelial cell receptors [26]. In 2012, Stapleton *et al.* demonstrated in a placebo controlled trial that women randomized to cranberry juice had a concurrent but non-significant reduction in numbers of P-fimbriated *E. coli* in urine and in the rate of symptomatic UTIs [27].

The optimal PAC concentration, dosage regimen, and formulation are not known. In early studies with cranberries the chemical composition of available cranberry products was not standardized, and the dose not properly described. In more recent studies PAC standardized cranberry products have been used.

Based on the studies from Stothers [28] and Kontiokari *et al.* [21] in a Cochrane review from 2008, it was concluded that cranberry products significantly reduced the incidence of UTIs at 12 months (RR 0.65, 95% CI 0.46–0.90) compared with placebo/control in women with recurrent UTIs. However, in a more recent version of this Cochrane review it was concluded that cranberry products did not significantly reduce the occurrence of symptomatic UTI in women with recurrent UTIs. This difference is due to the inclusion of two additional trials in their meta-analysis. However, one of these studies was in women with an acute (not recurrent) UTI [29], and the other study [30] determined the effect of cranberry consumption on the amelioration of symptoms rather than on the incidence of recurrences.

In two studies, the effectiveness of cranberry extract was compared with low-dose antibiotic prophylaxis. In a study with 137 older women with two or more antibiotic-treated UTIs in the previous 12 months, the authors concluded that TMP had a limited advantage over cranberry extract in the prevention of recurrent UTIs in older women [31]. In a more recent study, it was shown that cranberry capsules are less effective than low-dose (480 mg) TMP-SMX in the prevention of recurrent UTIs in premenopausal women. However, in contrast to low-dose TMP-SMX, cranberries did not result in an increase in resistant micro-organisms in the commensal flora [32].

#### 3.2.2. d-Mannose

*In vitro* and *in vivo* animal studies have shown that d-mannose can inhibit the adhesion of Type 1 fimbria of uropathogenic bacteria to the uroepithelial cells [33]. Recently, the first randomized clinical trial that evaluated its effectiveness was published [34]. Three hundred and eight women with recurrent UTIs were randomized to receive daily 2 grams of d-mannose prophylaxis for six months, continuous antibiotic prophylaxis with nitrofurantoin, or no prophylaxis. The rate of recurrent UTI in the d-mannose group was 15%, compared to 20% in the nitrofurantoin group, and 60% in the group without prophylaxis. These initial findings are promising, but further clinical trials are needed.

## 4. Future Prospects

### 4.1. Vaccination

Various bacterial extracts have been used in the management of recurrent UTIs. An effective bacterial extract must be able to stimulate the host’s immune system to produce antibodies and cytokines [35]. However, the exact mechanisms of protection and immunological basis remain unclear.

#### 4.1.1. Oral Immunostimulant OM-89

The oral immunostimulant OM-89, an extract of 18 different serotypes of heat-killed uropathogenic *E. coli,* stimulates innate immunity by increasing neutrophils, macrophage phagocytosis, and the upregulation of dendritic cells. Four placebo-controlled studies [36,37,38,39], with a total of 891 patients with recurrent UTIs, evaluating the oral immunostimulant OM-89 have been reported. During the intervention period women used one capsule daily. The risk ratio for developing at least one UTI was significantly lower in the active treatment group (RR 0.61, 95% CI 0.48–0.78) and the mean number of UTIs was about half compared to placebo. Despite these initial promising findings, a recent trial did not demonstrate a preventive effect of OM-89 compared to placebo. Remarkably, in the same trial a preventive effect of nitrofurantoin was also not observed. Since the preventive effect of nitrofurantion is well-established [1], the lack of effect of nitrofurantoin and OM-89 might be due to the low number of UTIs that occurred during the study [40].

#### 4.1.2. Vaginal Vaccine Urovac^®^

Urovac^®^ is a vaginal vaccine that contains 10 heat-killed uropathogenic bacterial species, including six different serotypes of uropathogenic *E. coli*, *Proteus vulgaris*, *Klebsiella pneumoniae*, *Morganella morganii,* and *Enterococcus faecalis*. This vaccine induces primarily immunoglobulin G and immunoglobulin A in the urogenital tract, thereby reducing potential colonization of the vagina and bladder with uropathogens [41]. In three trials comprising 220 women, all by the same group of investigators, placebo was compared with primary immunization [42], or with primary immunization with booster immunizations [41,43]. Primary immunization consisted of three vaginal vaccine suppositories at weekly intervals. Booster immunization consisted of three additional vaccine suppositories at monthly intervals. Primary immunization alone did not reduce UTI recurrence. However, following the booster immunizations there was a prolonged time to the first recurrence of UTI, compared to primary immunization only or placebo.

### 4.2. Acupuncture

Acupuncture has been used in the treatment and prevention of many clinical conditions. We identified two open trials [44,45], by the same group of investigators, on the use of acupuncture for prevention of recurrent UTIs in women. In the first trial, 67 women received real acupuncture, sham acupuncture, or no treatment. In the other trial, 94 women received acupuncture or no treatment. In the real acupuncture group needles were inserted to the correct depth at known acupuncture points, the qi sensation was obtained, and needles were manipulated by hand. In the sham acupuncture group needles were inserted superficially, outside known acupuncture points and without manipulation. All treatments were administered twice weekly for four weeks. Real acupuncture significantly reduced the proportion of women experiencing a UTI compared to no treatment (RR 0.48, 95% CI 0.29–0.79). The effect of sham acupuncture on the incidence of UTI was comparable to that of no treatment. Although these two studies suggest that acupuncture might prevent UTIs, these results should be interpreted with caution until confirmed by larger well-designed double-blind randomized trials. In addition, the mechanism of action remains unclear.

### 4.3. Gastrointestinal Decolonization of Multiresistant Bacteria

Transplant pyelonephritis caused by an extended spectrum beta-lactamase (ESBL) producing *E*. *coli* intestinal colonization is a problem in renal transplant patients. The decline in renal function with recurrent severe infection can result in end stage renal disease necessitating another renal transplantation. However, the risk for recurrent pyelonephritis by ESBL producing *E. coli* due to persistent colonization is a relative contraindication for another renal transplant procedure. Recently, the first case report was published of a patient with recurrent episodes of transplant pyelonephritis who was decolonized for ESBL-producing *E. coli* with a fecal microbiota transplantation [46]. Two weeks after fecal transplantation the rectal culture became ESBL negative and during the follow up the patient did not develop symptoms of a UTI.

## 5. Conclusions

Increasing antimicrobial resistance has stimulated interest in non-antibiotic prophylaxis of recurrent UTIs. Prophylaxis with non-antimicrobial agents does not result in an increase of antimicrobial resistance of the commensal flora. Therefore, the use of topical vaginal estrogen, oral capsules with *L. rhamnosus GR-1* and *L. reuteri RC-14* in post-menopausal women, and cranberry prophylaxis or intravaginal *Lactobacillus crispatus* in premenopausal women have been considered. Further research is needed to define the optimal dosage of cranberry products. In addition, further study of d-mannose, acupuncture, or vaccination is needed. Based on available evidence, ascorbic acid (vitamin C) cannot be recommended to prevent recurrences.
